# Establishment and validation a relapse prediction model for bipolar disorder

**DOI:** 10.3389/fpsyt.2024.1500892

**Published:** 2025-01-16

**Authors:** Xiaoqian Zhang, Minghao Wu, Daojin Wang, Long Wang, Wen Xie

**Affiliations:** ^1^ School of Mental Health and Psychological Science, Anhui Medical University, Hefei, China; ^2^ Department of Mood Disorder, Affiliated Psychological Hospital of Anhui Medical University, Hefei, China; ^3^ Department of Mood Disorder, Hefei Fourth People’s Hospital, Hefei, China; ^4^ Department of Mood Disorder, Anhui Mental Health Center, Hefei, China; ^5^ Department of Psychiatry, Wuhu Hospital of Beijing Anding Hospital, Capital Medical University (Wuhu Fourth People’s Hospital), Wuhu, China

**Keywords:** bipolar disorder, recurrence, risk factors, nomogram, prediction model

## Abstract

**Background:**

The recurrence rate of bipolar disorder (BD) is relatively high. Assessing the risk of relapse in patients with BD can assist in identifying populations at high risk for recurrence, and early feasible interventions can improve patient’ prognoses. Therefore, it is important to establish and validate predictive models for relapse risk in patients with BD.

**Method:**

We used 303 patients with BD admitted to the Anhui Mental Health Center as a retrospective training cohort and 81 patients from the Wuhu Fourth People’s Hospital as an external validation cohort. We collected a multidimensional assessment of the characteristics of patients eligible for enrollment, including general demographic characteristics, medical history, treatment, and assessment of selected scales. At the same time, they were followed up for 1 year after reaching the recovery standard after treatment. Depending on whether their symptoms returned within a year, patients with BD were divided into recurrent and non-recurrent groups. Recurrence risk factors for BD were selected using univariate and binary logistic regression analyses based on the clinical data of the patients and other pertinent information. A nomogram model was developed to predict the incidence of BD relapse. To further assess the model fit and dependability, calibration curves, working curves of subject attributes, and decision curves were also employed.

**Result:**

A total of 384 patients with BD were enrolled in this study, of whom 250(65.1%) had non-recurrent episodes and 134(34.9%) had recurrent episodes. Of these, 96 (31.7%) had relapses at the Anhui Mental Health Centre and 38 (46.9%) at the Fourth People’s Hospital of Wuhu City. According to the results of univariate and multivariate logistic regression analyses, the number of prior episodes (odds ratio [OR]: 1.38, 95% confidence interval [CI]: 1.179–1.615), Social Disability Screening Schedule (SDSS) score (OR: 1.303, 95% CI: 1.027-1.652), Pittsburgh Sleep Quality Index (PSQI) (OR: 1.476, 95% CI: 1.29-1.689), Number of visits(OR: 0.768, 95% CI: 0.684-0.863), suicidal behaviors (OR: 5.54, 95% CI: 1.818-16.881) and the electroconvulsive therapy (ECT) (OR: 0.382, 95% CI: 0.156-0.94) were independent risk factors for relapse in patients with BD. An analysis of the receiver operating characteristic curve, calibration curve, and clinical decision curve further revealed that the predictive efficiency and degree of fit between the predicted value of the nomogram and the actual observed value were better.

**Conclusion:**

This study found that the number of previous episodes, SDSS score, PSQI score and suicidal behaviors were independent risk factors for relapse of BD, while the number of visits and ECT were protective factor. Based on these factors, we developed and validated a nomogram for predicting relapse in patients with BD; that has clinical reference values.

## 1 Introduction

Bipolar Disorder (BD) is a mental disorder characterized by recurrent manic and depressive symptoms (1). BD is often associated with drug abuse, anxiety, suicide, and other diseases. The proportion of comorbidity is high, seriously impacting patient health and social stability (2, 3). Even if patients with BD, especially hospitalized patients, receive standardized treatment, the disease is characterized by recurrent episodes of mania, depression, or mixed states (4),which places enormous pressure on families and healthcare providers. The treatment effect in patients with BD is often difficult to maintain; therefore, the main clinical feature is symptom recurrence (5). Understanding the long-term response of patients to treatment and the predictive factors for disease recurrence helps clinicians and patients monitor treatment effectiveness and reduces direct and indirect treatment costs. Studies have reported that the annual recurrence rate of patients with BD varies from 39% to 52% during different treatment and maintenance plans (6). Therefore, the prevention and treatment of symptom recurrence are important for disease treatment.

Although the relapse rate in patients with BD is high, there is still a lack of effective clinical prediction models to predict the risk of relapse. However, developing nomogram can effectively help solve this problem; as it integrates several important factors and predicts whether the condition in these patients will relapse after active standardized treatment (7). Therefore, we conducted a retrospective study on patients with BD who received standardized treatment, collected information such as demographic characteristics and general clinical characteristics of patients, constructed and verified a risk prediction model of relapse within 1 year in patients with BD, and predicted the relapse risk of individual patients, to provide a certain reference value for clinical treatment response and prognosis judgment.

## 2 Materials and methods

### 2.1 Patients

In this study, 303 patients with bipolar disorder were retrospectively collected from March 2021 to May 2022 at Anhui Mental Health Centre. At the same time, we also collected 81 patients who met the enrolment criteria at the Fourth People’s Hospital of Wuhu City. Patients were followed up for 1 year after reaching the recovery standard after treatment.

#### 2.1.1 Inclusion criteria

(1) BD compliance with the International Classification of Diseases (ICD-10); (2) Unilateral seizures; (3) Age: 14-65 years; (4) A Hamilton Depression Scale (HAMD) (8)score or the Bech Rafaelsen Mania Rating Scale (BRMS) score on the Mania Rating Scale of >75% after treatment with administration of mood stabilizers and antipsychotic drugs; (5) A follow-up period of 1 year; (6) Presence of at least one informant who understands the patient’s situation;

#### 2.1.2 Exclusion criteria

(1) Patients with organic brain lesions or a history of major brain trauma; (2) Neurological disorders; (3) Concomitant conditions, such as schizophrenia and vascular dementia; (4) Illiteracy (inability to complete the questionnaire); (5) Visual, auditory, reading, and writing impairments; (6) Missing visit.

BD relapse was defined as: (1) Patient visit or hospitalization because of mood disorders; (2) Patients met the ICD-10 criteria for manic, hypomanic, or depressive episodes. Patients were divided into relapse and non-relapse groups based on whether they relapsed within 1 year of effective treatment.

The study was approved by the ethical committees of the Anhui Mental Health Centre (approval number: HFSY-IRB-YJ-LWTG-ZXQ( (2023-061-01)) and the Wuhu Fourth People’s Hospital (approval number:[2021]-KY-17).

### 2.2 Predictive variables and follow-up outcomes

This study collected patient-related information from patient’s medical records and questionnaires. Demographic characteristics and clinical variables including age, sex, duration of illness, and medications taken by the patients were assessed. The Pittsburgh Sleep Quality Index (PSQI) (9) and Social Disability Screening Schedule (SDSS) (10) scales were used to evaluate patients’ social function and sleep at discharge. In addition, we also collected multidimensional characteristics related to patients’ general demographic characteristics, past medical history, and treatment.

Meanwhile, we checked patients’ medical records or telephone follow-ups to investigate the recurrence of BD. We defined a positive outcome based on the recurrence of symptoms within 1 year of discharge for inpatients with BD and a negative outcome based on the lack of symptom recurrence.

### 2.3 Statistical method

In this study, we analyzed the data using SPSS 26.0 and R 4.3.1. The Kolmogorov-Smirnov test was used to determine the normal distribution of continuous variables, and the double sample t-test was used for normal distribution data, shown in the form of mean ± standard deviation. Non-parametric data were compared between groups using the Mann-Whitney U test and are presented as quartiles (P_25_–P_75_). For categorical variables, the chi-square test or Fisher’s exact test was employed to compare groups, and the results were expressed as frequency (%). This study used univariate regression analysis to analyze the predictive variables. To eliminate the impact of multicollinearity on the model, we perform covariance screening when including variables in the model. Variables with a Variance Inflation Factor (VIF) greater than 5 are addressed accordingly. The binary logistic regression included variables with p <0.05, and the regression prediction model was established and transformed into a nomogram model. A calibration curve, receiver operating characteristic (ROC) curve, decision curve analysis (DCA) curve and C-index were used to evaluate and verify the nomogram model. This study defined a significant difference as a two-tailed p < 0.05.

## 3 Results

### 3.1 Clinical characteristics

**Table 1** demonstrates the comparison of the relevant feature information between the training set and the external validation set. The training set included a total of 303 patients, of which 139 were female (45.9%) and 164 were male (54.1%). A total of 96 (31.7%) patients experienced recurrence and 207 (68.3%) did not. A total of 44 (54.3%) females and 37 (45.7%) males were included in the external validation center. A total of 38 (46.9%) patients in the external validation set experienced recurrence and 43 (53.1%) did not.

**Table 1:** Demographics and clinical characteristics of study in the training and validation set.

Variables	All data (n=384)	Training set (n=303)	Validation set (n=81)	t/Z/χ²	P-value
Age	33.74±12.3	33.07±11.569	36.23±14.529	-1.813	0.073
Hospital days	41.15±25.708	46.31±25.048	21.86±17.844	9.978	0.000
Disease duration (year)	9.11±8.382	8.585±7.646	11.074±10.52	-1.993	0.049
Age at onset	24.63±9.447	24.49±9.034	25.16±10.9	-0.513	0.609
Number of previous episodes	3.92±3.313	3.5±2.985	5.46±3.981	-4.114	0.000
Years of education	10.97±3.629	11.52±3.279	8.93±4.135	5.221	0.000
SDSS score	3.57±2.959	2.9±2.117	6.07±4.119	-6.7	0.000
PSQI score	7.07±3.838	6.57±3.58	8.93±4.212	-4.6	0.000
Number of visits	5.76±4.444	6.01±4.366	4.83±4.634	2.138	0.033
Sex				1.828	0.176
female	201 (52.3%)	139 (45.9%)	44 (54.3%)		
male	183 (47.7%)	164 (54.1%)	37 (45.7%)		
First onset type				1.181	0.277
mania	188 (49.0%)	144 (47.5%)	44 (54.3%)		
depression	196 (51.0%)	159 (52.5%)	37 (45.7%)		
Working status				3.325	0.068
yes	191 (49.7%)	158 (52.1%)	33 (40.7%)		
no	193 (50.3%)	145 (47.9%)	48 (59.3%)		
Marriage				5.236	0.073
unmarried	171 (44.5%)	139 (45.9%)	32 (39.5%)		
married	180 (46.9%)	143 (47.2%)	37 (45.7%)		
spouse or dissociation	33 (8.6%)	21 (6.9%)	12 (14.8%)		
Living state				4.694	0.03
living alone	25 (6.5%)	24 (7.9%)	1 (1.2%)		
non-living alone	359 (93.5%)	279 (92.1%)	80 (98.8%)		
Combined physical disease				6.025	0.014
yes	57 (14.8%)	38 (12.5%)	19 (23.5%)		
no	327 (85.2%)	265 (87.5%)	62 (76.5%)		
Mood stabilizers				27.105	0.000
none	4 (1.0%)	2 (0.7%)	2 (2.5%)		
one	303 (78.9%)	224 (73.9%)	79 (97.5%)		
two	76 (19.8%)	76 (25.1%)	0 (0%)		
three or more	1 (0.3%)	1 (0.3%)	0 (0%)		
Types of antipsychotic drugs				4.193	0.123
none	4 (1.0%)	4 (1.3%)	0 (0.0%)		
one	354 (92.2%)	275 (90.8)	79 (97.5%)		
two or more	26 (6.8%)	24 (7.9%)	2 (2.5%)		
Attempted suicide				0.118	0.731
yes	57 (14.8%)	44 (14.5%)	13 (16.0%)		
no	327 (85.2%)	259 (85.5%)	68 (84.0%)		
Combined mental symptoms				44.836	0.000
yes	151 (39.3%)	93 (30.7%)	58 (71.6%)		
no	233 (60.7%)	210 (69.3%)	23 (28.4%)		
MECT therapy				19.715	0.000
yes	103 (26.8%)	97 (32.0%)	6 (7.4%)		
no	281 (73.2%)	206 (68.0%)	75 (92.6)		
Outcome				6.526	0.011
relapse group	134 (34.9%)	96 (31.7%)	38 (46.9%)		
non-relapse group	250 (65.1%)	207 (68.3%)	43 (53.1%)		

SDSS, social disability screening schedule; PSQI, pittsburgh sleep quality index; MECT, modified electroconvulsive therapy.

**Table 2** demonstrates the comparison between the baseline characteristics of the relapse and non-relapse groups of patients with BD in the training set. In the relapse group, the duration of disease was significantly higher than in the non-relapse group (10.313 ± 8.68 vs. 7.784 ± 6.994, p=0.007). Age at onset was significantly lower in the relapse group than in the non-relapse group (22.19 ± 7.32 vs 25.55 ± 9.556, p=0.002). The number of previous episodes was also significantly higher in the relapse group than in the non-relapse group (4.77 ± 3.922 vs. 2.92 ± 2.209, p=0.000). The number of years of education was also significantly higher in the non-relapse group than in the relapse group (11.81 ± 3.319 vs 10.89 ± 3.115, p=0.022). In the comparison of SDSS scores, it was significantly higher in the relapse group than in the non-relapse group (4.33 ± 2.405 VS 2.24 ± 1.582, p=0.000). In the comparison of PSQI scores, the relapse group was significantly higher than the non-relapse group (9.45 ± 3.808 VS 5.24 ± 2.543, p=0.000). In the comparison of the number of recurrence visits, the non-recurrence group was significantly higher than the recurrence group (7.03 ± 4.463 VS 3.81 ± 3.203, p=0.000). In the relapse group, the percentage of patients with suicidal behavior was significantly higher than in the non-relapse group (24.0% vs 10.1%, p=0.001). In addition, the proportion of ECT was significantly higher in the non-relapse group than in the relapse group (35.7% vs 24.0%, p=0.041).

**Table 2:** Comparison of demographic and clinical characteristics of the relapse and no-Relapse groups of BD patients in the training set.

Variables	All data (n=303)	Relapse group (n=96)	Non-relapse group (n=207)	t/Z/χ²	P-value
Age	33.07±11.569	32.52±11.8	33.32±11.48	-0.561	0.575
Hospital days	46.31±25.048	45.63±22.8	46.62±26.069	-0.322	0.747
Disease duration (year)	8.585±7.646	10.313±8.68	7.784±6.994	2.706	0.007
Age at onset	24.49±9.034	22.19±7.32	25.55±9.556	-3.056	0.002
Number of previous episodes	3.5±2.985	4.77±3.922	2.92±2.209	4.322	0.000
Years of education	11.52±3.279	10.89±3.115	11.81±3.319	-2.303	0.022
SDSS score	2.9±2.117	4.33±2.405	2.24±1.582	7.796	0.000
PSQI score	6.57±3.58	9.45±3.808	5.24±2.543	9.852	0.000
Number of visits	6.01±4.366	3.81±3.203	7.03±4.463	-7.137	0.000
Sex				0.057	0.812
female	139 (45.9%)	45 (46.9%)	94 (45.4%)		
male	164 (54.1%)	51 (53.1%)	113 (54.6%)		
First onset type				0.803	0.37
mania	144 (47.5%)	42 (43.8%)	102 (49.3%)		
depression	159 (52.5%)	54 (56.3%)	105 (50.7%)		
Working status				15.759	0.000
yes	158 (52.1%)	34 (35.4%)	124 (59.9%)		
no	145 (47.9%)	62 (64.6%)	83 (40.1%)		
Marriage				5.236	0.073
unmarried	139 (45.9%)	48 (50.0%)	91 (44.0%)		
married	143 (47.2%)	43 (44.8%)	100 (48.3%)		
spouse or dissociation	21 (6.9%)	5 (5.2%)	16 (7.7%)		
Living state				4.04	0.044
living alone	24 (7.9%)	12 (12.5%)	12 (5.8%)		
non-living alone	279 (92.1%)	84 (87.5%)	195 (94.2%)		
Combined physical disease				0.578	0.447
yes	38 (12.5%)	10 (10.4%)	28 (13.5%)		
no	265 (87.5%)	86 (89.6%)	179 (86.5%)		
Mood stabilizers				1.99	0.000
none	2 (0.7%)	0 (0%)	2 (1.0%)		
one	224 (7.9%)	69 (71.9%)	155 (74.9%)		
two	76 (25.1%)	27 (28.1%)	49 (23.7%)		
three or more	1 (0.3%)	0 (0%)	1 (0.4%)		
Types of antipsychotic drugs				1.984	0.371
none	4 (1.3%)	2 (2.1%)	2 (1%)		
one	275 (90.8%)	89 (92.7%)	186 (89.9)		
two or more	24 (7.9%)	5 (5.2%)	19 (9.1%)		
Attempted suicide				10.082	0.001
yes	44 (14.5%)	23 (24.0%)	21 (10.1%)		
no	259 (85.5%)	73 (76.0%)	186 (89.9%)		
Combined mental symptoms				0.169	0.681
yes	93 (30.7%)	31 (32.3%)	62 (30.0%)		
no	210 (69.3%)	65 (67.7%)	145 (70.0%)		
MECT therapy				4.189	0.041
yes	97 (32.0%)	23 (24.0%)	74 (35.7%)		
no	206 (68.0%)	73 (76.0%)	133 (64.3%)		

SDSS, social disability screening schedule; PSQI, pittsburgh sleep quality index; MECT, modified electroconvulsive therapy.

### 3.2 Results of univariate regression analysis

The results of univariate regression analysis showed that disease duration (odds ratio [OR]: 1.042, p = 0.009), age at onset (OR: 0.953, p = 0.003), number of previous episodes (OR: 1.233, p = 0.03), years of education(OR: 0.915, p = 0.023), SDSS score (OR: 1.75, p = 0.000), PSQI score(OR: 1.499, p = 0.000), Number of visits(OR: 0.768, p = 0.000), working status(OR: 0.367, p = 0.000), housing conditions(OR: 2.321, p = 0.049), mood stabilizer(p = 0.000), suicidal behaviors(OR: 2.791, p = 0.002) and ECT(OR: 0.566, p = 0.042) were the risk factors for relapse in patients with BD, with statistically significant differences (all p < 0.05) (**Table 3**).

**Table 3:** Univariable regression analysis of the various factors and relapse outcome.

Variables	Partial regression coefficient	Standard error	Wald value	OR	95%CI	P-value
Age	-0.006	0.011	0.317	0.994	0.973-1.015	0.994
Hospital days	-0.002	0.005	0.104	0.998	0.989-1.008	0.747
Disease duration (year)	0.042	0.016	6.869	1.042	1.011-1.075	0.009
Age at onset	-0.048	0.016	8.681	0.953	0.923-0.984	0.003
Number of previous episodes	0.209	0.046	20.411	1.233	1.126-1.35	0.000
Years of education	-0.088	0.039	5.173	0.915	0.848-0.988	0.023
SDSS score	0.56	0.079	49.87	1.75	1.498-2.044	0.000
PSQI score	0.405	0.053	57.694	1.499	1.35-1.664	0.000
Number of visits	-0.215	0.038	31.939	0.806	0.748-0.869	0.000
Sex						
male	reference					
female	0.059	0.248	0.057	1.061	0.653-1.723	0.812
First onset type						
mania	reference					
depression	0.222	0.248	0.802	1.249	0.768-2.032	0.371
Working status						
no	reference					
yes	-1.002	0.256	15.299	0.367	0.222-0.607	0.000
Marriage						
unmarried	reference					
married	-0.204	0.255	0.641	0.815	0.494-1.344	0.423
spouse or dissociation	-0.523	0.543	0.931	0.592	0.205-1.716	0.335
Living state						
non-living alone	reference					
living alone	-0.842	0.429	3.861	2.321	1.002-5.378	0.049
Combined physical disease						
no	reference					
yes	-0.297	0.391	0.575	0.743	0.345-1.6	0.448
Mood stabilizers						
none	reference					
one	20.394	28421.593	0.000	719168341	0-+∞	0.000
two	20.607	28421.593	0.000	890186190	0-+∞	0.000
three or more	0.000	49226.637	0.000	1	0-+∞	0.000
Types of antipsychotic drugs						
none	reference					
one	-0.737	1.008	0.534	0.478	0.066-3.452	0.478
two or more	-1.335	1.119	1.423	0.263	0.029-2.36	0.263
Attempted suicide						
no	reference					
yes	1.026	0.332	9.56	2.791	1.456-5.348	0.002
Combined mental symptoms						
no	reference					
yes	0.109	0.266	0.169	1.115	0.662-1.878	0.681
MECT therapy						
no	reference					
yes	-0.569	0.28	4.135	0.566	0.327-0.98	0.042

SDSS, social disability screening schedule; PSQI, pittsburgh sleep quality index; MECT, modified electroconvulsive therapy.

### 3.3 Multivariate regression analysis results

Multivariate regression analyses showed that the number of previous episodes (OR: 1.38, p = 0.000), Number of visits (OR: 0.806, p = 0.000), SDSS score (OR: 1.303, p = 0.029), PSQI scores (OR: 1.476, p = 0.000), suicidal behaviors (OR: 5.54, p = 0.003) and ECT (OR: 0.382, p =0.036) were statistically significant independent risk factors for relapse within 1 year in patients with BD (all p < 0.05) (**Table 4**).

**Table 4:** Multivariate regression analysis of the various factors and relapse outcome.

Variables	Partial regression coefficient	Standard error	Wald value	OR	95%CI	P-value
Disease duration(year)	0.003	0.032	0.011	1.003	0.943-1.068	0.916
Age at onset	-0.047	0.026	3.328	0.954	0.907-1.003	0.068
Number of previous episodes	0.322	0.08	16.048	1.38	1.179-1.615	0.000
Years of education	-0.051	0.065	0.606	0.951	0.837-1.08	0.436
SDSS score	0.265	0.121	4.764	1.303	1.027-1.652	0.029
PSQI score	0.389	0.069	32.172	1.476	1.29-1.689	0.000
Number of visits	-0.264	0.059	19.856	0.768	0.684-0.863	0.000
Working status						
no	reference					
yes	-0.276	0.458	0.361	0.759	0.309-1.864	0.548
Living state						
non-living alone	reference					
living alone	1.19	0.672	3.138	3.288	0.881-12.271	0.076
Mood stabilizers						
none	reference					
one	18.951	22479	0.00	169995823	0- +∞	0.999
two	19.493	22479	0.00	292282171	0- +∞	0.999
three or more	-0.49	46051	0.00	0.613	0- +∞	1
Attempted suicide						
no	reference					
yes	1.712	0.569	9.067	5.54	1.818-16.881	0.003
MECT therapy						
no	reference					
yes	-0.961	0.459	4.387	0.382	0.156-0.94	0.036

SDSS, social disability screening schedule; PSQI, pittsburgh sleep quality index; MECT, modified electroconvulsive therapy.

### 3.4 Development of the nomogram model

**Figure 1:**
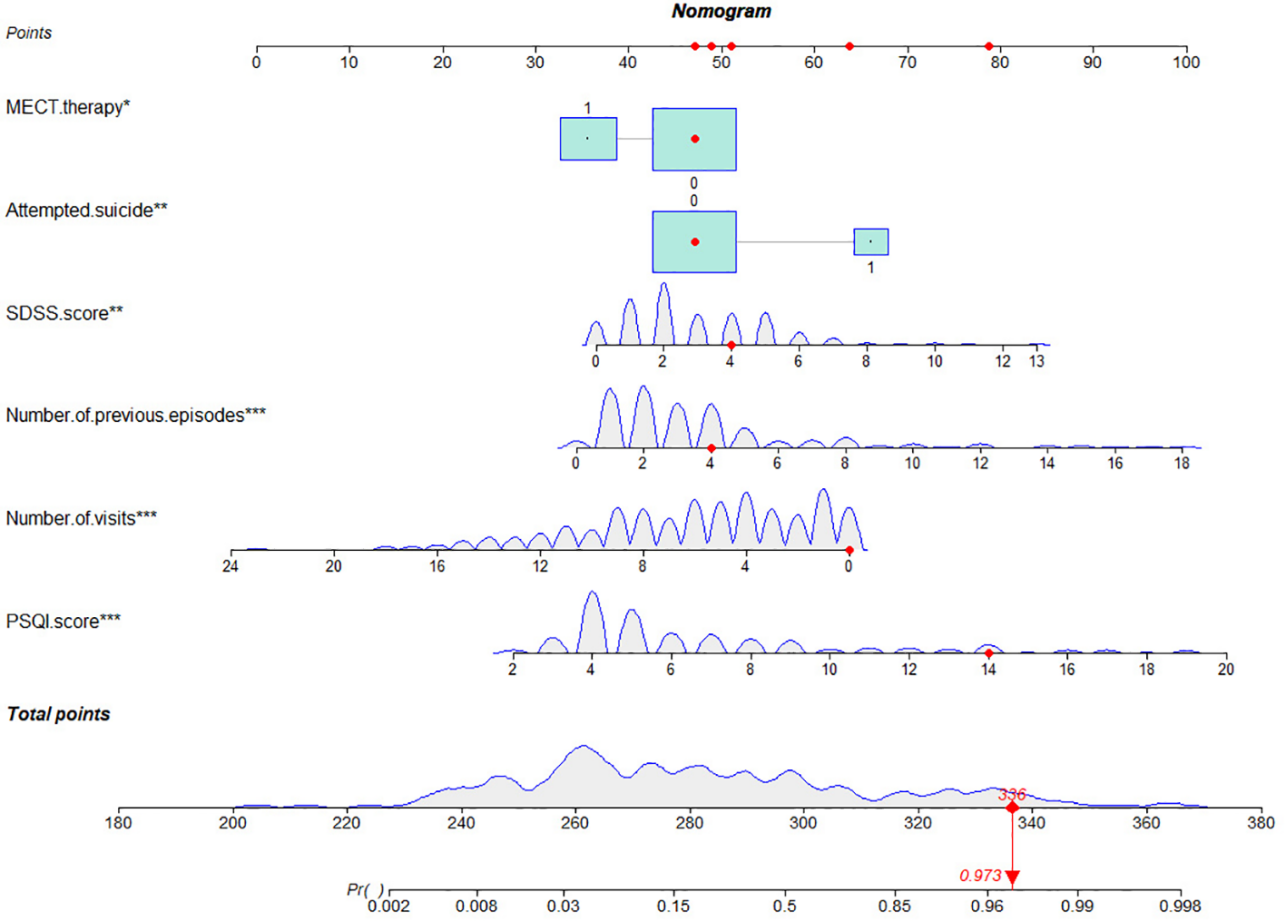
Nomogram for predicting the disease relapse of BD patients. For MECT therapy, 0=no, 1=yes. For attempted suicide, 0=no, 1=yes. SDSS, social disability screening schedule; PSQI, pittsburgh sleep quality index; MECT, modified electroconvulsive therapy. *, **, ***: indicates statistical significance in multivariate regression analysis.

Based on the logistic regression analyses described above, we developed a nomogram model to predict the risk of recurrence within 1 year in patients with BD (**Figure 1**). Each clinical factor corresponds to a specific score, and a straight- line point axis was drawn to calculate the total score, which corresponds to a high risk of BD relapse and vice versa.

### 3.5 Validation of the nomogram model

**Figure 2:**
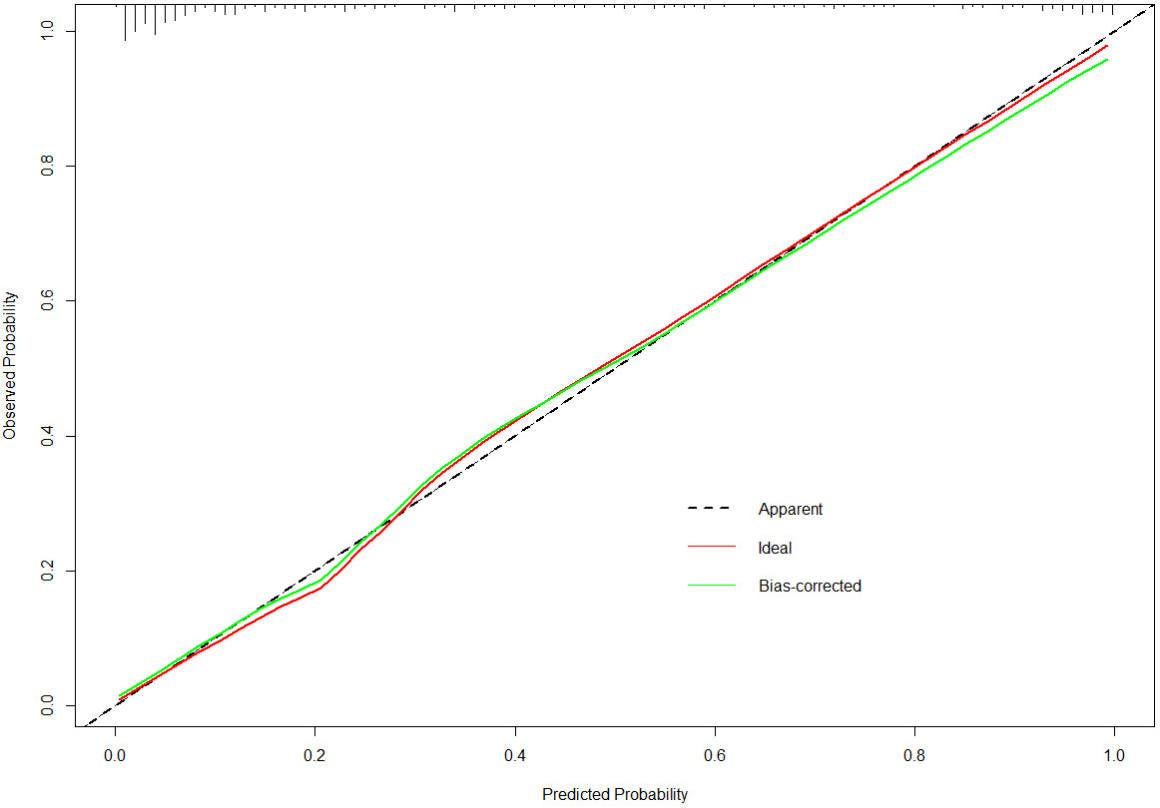
The calibration curves for the nomogram in the training set.

**Figure 3:**
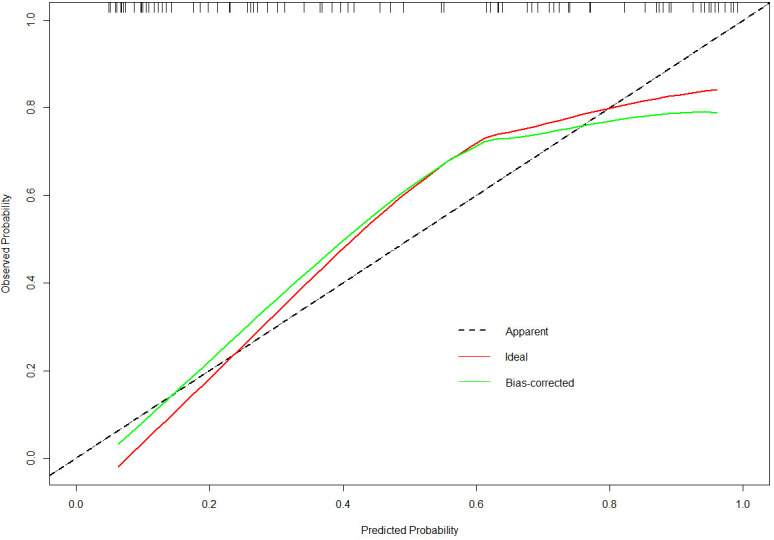
The calibration curves for the nomogram in the validation set.

**Figure 4:**
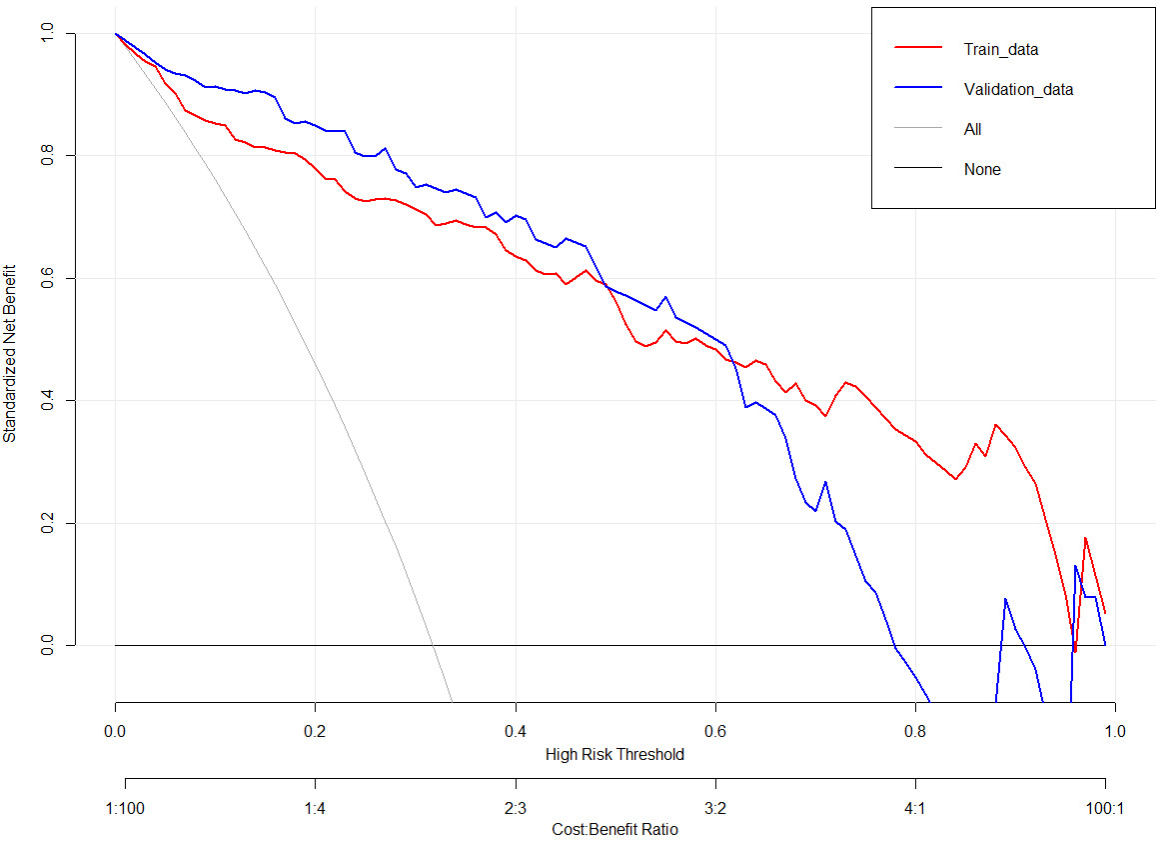
Decision curves for the clinical nomogram in the training and validation set.

**Figure 5:**
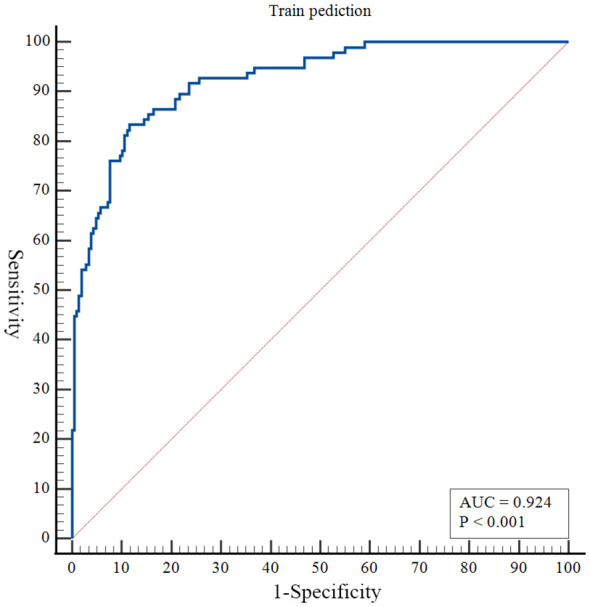
Receiver operating characteristic curves to evaluate the discriminating capability of the nomogram in the training set.

**Figure 6:**
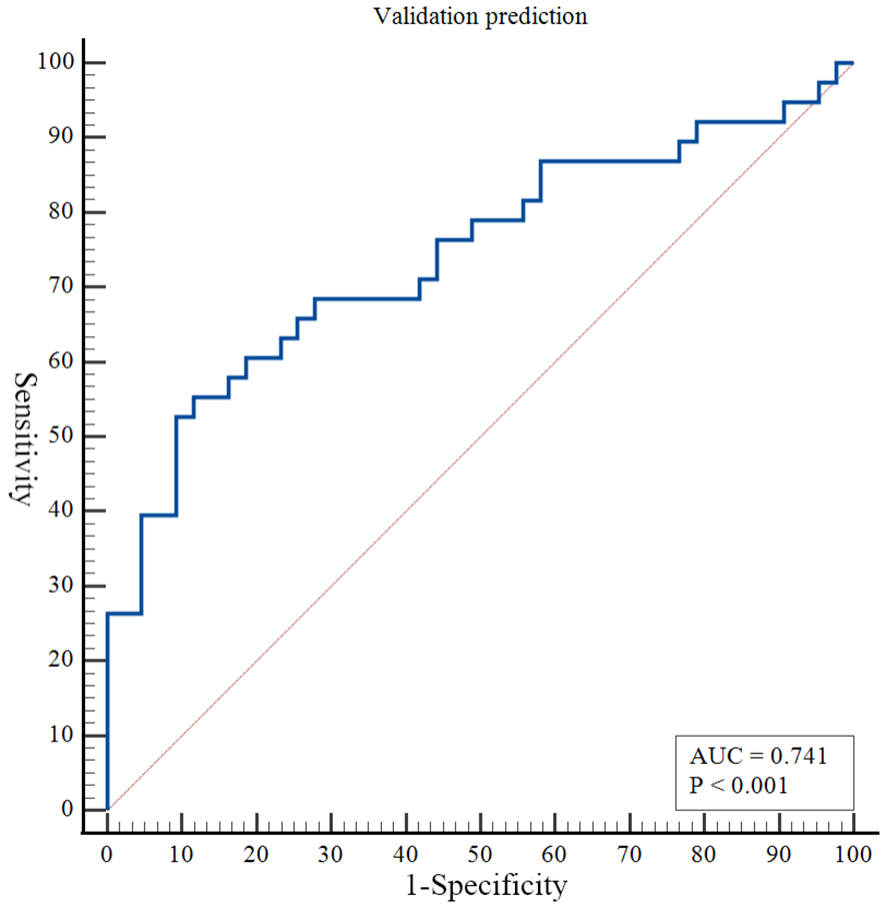
Receiver operating characteristic curves to evaluate the discriminating capability of the nomogram in the validation set.

The nomogram developed in this study have high predictive performance. Calibration curves were plotted from the predicted values of the nomogram model to the actual values. The results in **Figures 2**, **3** show that the agreement between the predicted and actual values of the nomogram model is high for both the training set and the validation set. In addition, **Figure 4** shows that the clinical prediction curves (DCA) have high clinical applicability on both the training set and the validation set. As shown in **Figure 5**, the area under the ROC curve suggests that the nomogram has high classification and prediction ability in the training set (AUC: 0.924, 95% CI: 0.888 - 0.951). **Figure 6** suggests that this nomogram model also has good generalization ability on the external test set (AUC:0.741, 95% CI:0.632 - 0.832).

## 4 Discussion

The high prevalence and relapse rate of BD place great pressure on the rehabilitation and treatment of patients (11). Therefore, establishing a risk prediction model for short-term recurrence is significance for early intervention and treatment guidance in patients with BD. In this study, 384 patients with BD were retrospectively included to analyze the risk factors that may affect short-term recurrence. Based on binary logistic regression analysis, this study found that the number of previous episodes, SDSS score, PSQI score, number of visits, suicidal behaviors and ECT were independent risk factors for short-term relapse in patients with this disease. Nomograms created by combining the above risk factors have a high predictive efficacy. Meanwhile, the performance of the external validation set demonstrated that the nomogram has a good panchromatic ability. The results showed that the model had a high predictive accuracy and clinical application value.

Recurrent relapses of symptoms in patients with BD can further deteriorate the condition and lead to poor prognosis (12), highlighting the importance of identifying factors associated with an increased risk of relapse in patients. Patients with BD have a large heterogeneity in the disease course and symptoms, and it is crucial to develop and validate relapse prediction models and guide individualized treatment. The results of multivariate logistic regression analysis showed that t the number of previous episodes, SDSS score, PSQI score, number of visits, suicidal behaviors and ECT were independent risk factors for relapse within 1 year.

Previous studies have shown that the number of previous episodes of a patient’s symptoms is an important predictor of relapse, which is consistent with our findings (13). On the one hand, prolonged depressive or manic episodes may disrupt the balance of neurotransmitters in the brain, such as dopamine, norepinephrine, and serotonin, making patients more susceptible to recurrent mood swings (14, 15). On the other hand, frequent symptomatic episodes can lead to cognitive decline, particularly impairments in memory, attention, and executive functioning, and may also make it more difficult for patients to effectively recognize and manage early signs of symptoms, thereby increasing the risk of relapse (16). Moreover, the results of this study indicate that the higher the number of previous episodes, the higher the risk of recurrence, and the higher the number of episodes, the more severe and complex the patient’s condition is, which in turn causes the patient’s disease to prolong, thus increasing the patient’s risk of recurrence (17). Our study also found that the number of outpatient visits was a protective factor against relapse in patients with BD. We found that the higher the number of outpatient follow-up visits, the lower the risk of relapse. Patients seek timely medical help based on dynamic changes in clinical symptoms; in contrast, doctors provide standardized medical management, suggesting the importance of patient compliance and standardized outpatient management. In addition, this study found that the higher the PSQI score, the higher the risk of recurrence; the worse the sleep quality, the higher the risk of BD recurrence. Some studies have found that sleep disorders may be precursors of BD (18). Some studies have confirmed that sleep disorders can affect circadian rhythms, change brain electrical activity during sleep, and impair patients’ emotional processing ability (19). Therefore, early maintenance of a good sleep state is of great value for the prognosis of patients with BD. Similarly, as SDSS scores increase, the risk of relapse increases in patients with BD. The SDSS scale is an instrument that responds to social and occupational functioning, and as the SDSS score increases, the poorer the social functioning of the respondent. Previous studies have shown that social dysfunction significantly increases the risk of relapse in patients with bipolar disorder (20).

The present study also found that the presence of suicidal behavior in patients significantly increased the risk of recurrence. It is well known that suicidal behavior usually responds to a more severe stage of illness in people with bipolar disorder, which significantly increases the risk of fluctuations and relapses (21). Previous studies have shown that suicidal behavior is closely associated with damage to the frontal lobe, which plays a critical role in emotion regulation, decision-making, and impulse inhibition (22). A reduction in frontal lobe function in patients exhibiting suicidal behavior may impair their ability to regulate negative emotions and control impulsive actions, thereby heightening the risk of relapse (23). This study also found that patients treated with ECT significantly reduced the risk of relapse in patients with bipolar disorder. ECT can rapidly relieve severe depressive and manic symptoms in patients with bipolar disorder, thereby stabilizing mood and reducing the likelihood of relapse (24). ECT is equally effective in patients with refractory BD, further stabilizing symptoms and thereby significantly reducing the risk of relapse (25).

However, even if patients with BD have the same or similar risk factors, the predicted risk of symptom recurrence is different. The combined prediction of multiple risk factors can improve predictive performance. Nomograms can combine multiple risk factors for joint prediction and have been widely used in statistical analyses of various neurological disorders (26). However, nomograms are rarely used to assess the risk of BD recurrence. In this study, we recruited 384 patients with BD and constructed a prediction model for symptom recurrence within 1 year. According to the multivariate logistic regression analysis results, a line chart scoring system was constructed based on the previous episodes, SDSS score, PSQI score and suicidal behaviors, the number of visits and ECT. The score system has a high predictive efficiency, with a AUC of 0.924 (95% CI: 0.888 - 0.951). Moreover, in order to test the generalisation ability of this nomogram model in this study, the prediction performance of the external test set was kept high (AUC:0.741, 95% CI:0.632 - 0.832). In addition, the calibration curve suggested a high degree of consistency between the predicted and actual values. The model aligns with actual clinical scenarios; in clinical settings, doctors can use column-line graphs to visualize the risk of relapse in patients with BD. In addition, combining the individual specificity of patients for early and comprehensive interventions has greater clinical value for the prognosis of patients with BD.

This study had some limitations. Firstly, this was a retrospective study. In the future, we will conduct a prospective cohort study to improve the reliability and persuasiveness of the study. Secondly, we established a prediction model based on demographic and clinical characteristics, and the input characteristics were relatively simple. In the future, we will further develop multigroup, multi-model, and multidimensional models to improve the model’s accuracy and clinical practice value. Finally, we did not use more cutting-edge AI techniques applied in this study, such as machine learning, deep learning, etc. In the future, we will actively explore the performance of high-performance AI techniques applied in the occurrence and development of BD. Finally, the relatively small sample size may limit the performance and generalizability of the model. In the future, we plan to conduct a multicenter study to expand the sample size and further validate our findings.

## 5 Conclusion

This study found that the number of previous episodes, SDSS score, PSQI score and suicidal behaviors were independent risk factors for relapse of BD, while the number of visits and ECT were protective factor. Based on these factors, we developed and validated a nomogram for predicting relapse in patients with BD; that has clinical reference values.

## Data Availability

The original contributions presented in the study are included in the article/supplementary material. Further inquiries can be directed to the corresponding author.
